# *Pectobacterium versatile* β‐Lactamase Contributes to Soft Rot *Pectobacteriaceae* (SRP) Community Diversity During Potato Infection

**DOI:** 10.1111/1758-2229.70111

**Published:** 2025-05-26

**Authors:** Camille Lorang, Pierre‐Yves Canto, Erwan Gueguen, Jacques Pédron, Marie‐Anne Barny

**Affiliations:** ^1^ INRAE, CNRS, Institut d'Ecologie et des Sciences de l'Environnement de Paris Sorbonne Université Paris France; ^2^ CNRS, UCBL, INSA, UMR5240 Microbiologie, Adaptation et Pathogénie Université de Lyon Villeurbanne France

**Keywords:** ecology, Pectobacteriaceae, species complex, β‐lactamase

## Abstract

Little is known about the role of antibiotics in microbial ecosystems in the absence of clinical antibiotic pressure. The soft rot *Pectobacteriaceae* (SRP) species complex comprises 37 bacterial species that are collectively responsible for severe plant decay in many crops. Within this complex, *Pectobacterium versatile* strains harbour the Bla_PEC‐1_ β‐lactamase. The aim of our work was to analyse the role of BlaPEC‐1 during plant infection. To this end, two *bla*_PEC‐1_‐deleted strains were compared with their wild‐type counterparts in vitro and in mono‐ or mixed infections of potato tubers with different SRP strains. In vitro, Bla_PEC‐1_ enables *P. versatile* to resist ampicillin or the carbapenem produced by *Pectobacterium brasiliense*. In mono‐infections of potato tubers, *bla*_PEC‐1_‐deleted strains were not affected in virulence, fitness, or association with bacterial commensals. In mixed infections, Bla_PEC‐1_ was required for the coexistence of *P. versatile* with the carbapenem‐producing strain and was necessary to rescue carbapenem‐sensitive strains both in vitro and *in planta*. Protection was observed even if both the *bla*_PEC‐1_ gene was repressed and the Bla_PEC1_‐expressing bacteria were a minority in the symptoms. These results indicate that Bla_PEC_‐1 exerts a true β‐lactamase function during the infection process and acts as a public good of the SRP species complex to maintain SRP strain diversity.

## Graphical abstract

This work analyses the ecological role exerted by β‐lactamase under natural conditions, in the absence of clinical antibiotic pressure. It shows that β‐lactamase acts as a common good and plays an important role in the maintenance of β‐lactam‐susceptible strains in the environment.

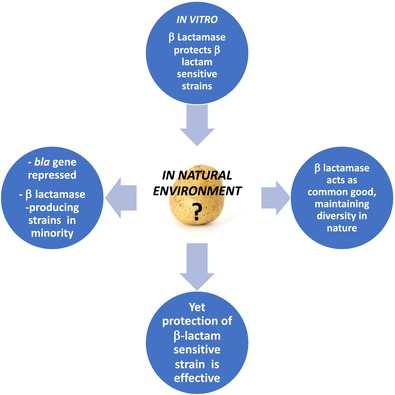

## 1 Introduction

Antibiotics are probably one of the most successful forms of chemotherapy in the history of medicine. The β‐lactam antibiotics, which disrupt bacterial cell wall formation in both Gram‐negative and Gram‐positive bacteria, are currently the most widely used class of antibacterial agents in the treatment of infectious diseases (Bush and Bradford 2016). All β‐lactam antibiotics share the β‐lactam ring as the essential core structure and are classified into four classes: penicillins, cephalosporins, carbapenems, and monobactams (Kim et al. 2023). The massive use of β‐lactams in human and animal health is leading to the constant emergence of bacterial resistance. In particular, the regular emergence of new β‐lactamases that cleave and inactivate different β‐lactam rings is a cause for concern (Tooke et al. 2019). To date, more than 2700 unique β‐lactamases have been described, and they are classified into four molecular classes (classes A to D) based on their primary structure, which are further subdivided into functional groups based on their substrate preferences (Bush 2018). For example, carbapenems are not hydrolyzed by most β‐lactamases, except class B and class D2df β‐lactamases. It is recognised that β‐lactamases are ancient enzymes that have existed without the pressure of therapeutic antibiotics and that they have emerged in clinically pathogenic species following transfer from environmental sources. The rapid spread of transmissible β‐lactamases carried on plasmids or transposons that hydrolyze recently approved cephalosporins, monobactams, and carbapenems is important in Gram‐negative clinical pathogens (Cantón et al. 2008). However, the spread of resistance by mobile genetic elements contrasts with the lack of knowledge about the evolutionary and ecological processes that take place in microbial ecosystems in the absence of antibiotic pressure. In natural ecosystems, antibiotics are not present at therapeutic doses, and some authors have proposed that antibiotics, in this context, act as regulatory or signalling molecules rather than weapons (Romero et al. 2011). This scenario is supported by in vitro analysis of the role of antibiotics at sub‐inhibitory concentrations but is poorly supported by experimental data in nature. To better understand the role and function of β‐lactam and β‐lactamases in natural ecosystems, it is important to develop experimental setups to study their role in the absence of clinical antibiotic selection pressure.

Soft rot *Pectobacteriaceae* (SRP) are an interesting model to analyse the role of β‐lactam and β‐lactamases in natural ecosystems. SRP are plant pathogens belonging to the genera *Pectobacterium* and *Dickeya* within the order Enterobacterales. SRP disrupt the plant cell wall and induce rot symptoms in infected host plants (Hugouvieux‐Cotte‐Pattat et al. 2014; Charkowski 2018). SRP can infect important crops such as potatoes, causing a disease called blackleg in potato fields and a rotting of potato tubers called soft rot, which can lead to severe post‐harvest losses. SRP form a species complex and several species are often associated within symptomatic or asymptomatic plant tissue (Degefu 2021; de Werra et al. 2021; Ge et al. 2021; Motyka‐Pomagruk et al. 2021; Smoktunowicz et al. 2022; Barny et al. 2024). Recently, a species, *Pectobacterium versatile*, was described to harbour a β‐lactamase, Bla_PEC‐1_, which is the closest relative of Bla_TEM‐1_, a class A2b β‐lactamase plasmid‐borne and widespread in clinical enterobacteria (Bush 2018; Royer et al. 2022). The oldest identified *P. versatile* strain carrying the Bla_PEC‐1_ β‐lactamase was isolated from *Solanum tuberosum* in Canada in 1918, prior to the discovery of the first β‐lactam antibiotic in the early 20th century, ruling out the possibility that the acquisition of this gene within the chromosome is associated with the clinical use of β‐lactam antibiotics (Royer et al. 2022). Phenotypic characterisation in vitro revealed a low level of *P. versatile* Bla_PEC‐1_ β‐lactamase expression and it remains unclear whether Bla_PEC‐1_ acts as a true β‐lactamase during the infection process or whether it is merely a signalling molecule as previously proposed (Romero et al. 2011; Royer et al. 2022). Interestingly, within symptomatic plants, *P. versatile* is often associated with other SRP species such as *Pectobacterium carotovorum* or *Pectobacterium brasiliense* and some strains of these latter species produce the structurally simplest member of the carbapenem class of β‐lactam antibiotics, the (5R)‐carbapen‐2‐em‐3‐carboxylic acid (Parker et al. 1982; McGowan et al. 1997, 2005).

The aim of the present work was to analyse the role of the *P. versatile* β‐lactamase Bla_PEC‐1_ in the context of plant infection with or without carbapenem‐producing *P. brasiliense* . To this end, we constructed *bla*_PEC‐1_‐deleted strains in two different *P. versatile* strain backgrounds and compared them with their wild‐type counterparts in vitro and *in planta*, with mono‐ or mixed infections with different *P. brasiliense* strains.

## 2 Experimental Procedures

### 2.1 Bacterial Strains, Plasmids and Growth Conditions

The bacterial strains, plasmids, and oligonucleotides used in this study are described in Tables S1 and S2 respectively. Briefly, the following strains were used in this study: *P. brasiliense* type strain CFBP6617 (hereafter 6617; other names 1692, BAA‐417, BPBB‐212, Ecbr‐212) (Liu and Filiatrault 2020), *P. brasiliense* CFBP5381 (hereafter 5381), *P. versatile* type strain CFBP6051 (hereafter 6051; other names ICMP9168, NCPPB3387, de Boer 21) (Portier et al. 2019) and its Δ*bla*_PEC‐1_ mutant derivative CL1, *P. versatile* A73‐S18‐O15 (hereafter A73)(Ben Moussa et al. 2022) and its Δ*bla*_PEC‐1_ mutant derivative CL2, *E. coli* DH5‐a‐λ‐pir and *E. coli* MFD‐pir. The strains were routinely grown at 30°C in LB (10 g/L tryptone, 5 g/L yeast extract, NaCl 5 g/L), in TSB (1.7 g/L casein peptone, 0.3 g/L soy peptone, 0.3 g/L NaCl, 0.25 g/L dipotassium hydrogen phosphate, 0.25 g/L glucose) or in CVP (1.02 g/L CaCl_2_, 5 g trisodium citrate, 2.0 g/L NH_4_NO_3_, 4 g/L agar, 2.8 mL NaOH 5 M, 18 g/L pectin Dipecta‐AG366‐Agdia‐Biofords‐USA) prepared as described by Ben Moussa et al. 2023 for monolayer CVP. Antibiotics were added as needed at the following concentrations: ampicillin (Amp), 200 μg/L; chloramphenicol (Cm), 50 μg/L; diaminopimelic acid (DAP) (57 μg/mL) was added for the growth of the *E. coli* MFD‐pir strain. The media were solidified with 15 g/L agar as needed.

### 2.2 Construction of Δbla_pec‐1_ Mutants

To construct in‐frame deletion mutants of the *bla*_pec‐1_ gene, a counter‐selection method was used (Edwards et al. 1998). The cloning vector pRE112, which is an R6K‐based suicide plasmid carrying the *sac*B gene and the *cat* gene (CmR) was used to clone into *Sac*I/*Kpn*I digested pRE112 two PCR fragments corresponding to the upstream and downstream 0.5‐kbp DNA of the *bla*_pec‐1_ gene using the T5 exonuclease‐dependent assembly method (Xia et al. 2019).

Chemically ultra‐competent DH5α λpir cells were prepared with the Mix & Go! *E. coli* Transformation Kit according to standard procedures (Zymo Research). Transformants were selected on LB plates supplemented with Cm. Colonies with the correct plasmid were selected by colony PCR using oligo pairs L762/L763 and DreamTaq DNA polymerase (Thermofisher, Waltham, MA, USA). Plasmids were extracted using the NucleoSpin Plasmid Kit (Macherey‐Nagel), verified by restriction digestion (NEB) and sequenced (Eurofins). The plasmids were then transferred into the competent *E. coli* strain MFDpir (Ferrières et al. 2010) which was prepared as described above for DH5α λpir. *E. coli* MFDpir produces the RP4 conjugation machinery, which allows the transfer of the suicide plasmid into *Pectobacterium* strains by conjugation. For conjugation, a few colonies of *P. versatile* A73 or *P. versatile* 6051 were mixed in the same proportion with MFDpir colonies carrying the plasmid of interest in 500 μL LB and centrifuged at 6000 rpm for 2 min. The pellet was resuspended in 90 μL LB containing 5 μL DAP at 57 mg/mL and plated onto an LB agar plate incubated at 28°C. After 24 h, the bacteria were resuspended in 1 mL LB, diluted in 10‐fold series from 10^−1^ to 10^−7^, and plated on LB agar supplemented with Cm at 4 μg/L to select the first recombination event. Transconjugants re‐isolated on this medium were then plated on LB agar supplemented with 5% sucrose and incubated at 19°C for 2–3 days to allow the second recombination event. Sucrose‐resistant colonies were then streaked onto LB‐Cm plates to check for plasmid loss and onto LB‐Amp plates to check for *bla*_pec‐1_ gene loss. Total DNA from sucrose‐resistant, Cm and Amp‐sensitive colonies was used to amplify the mutagenized locus using Prime start max DNA polymerase (Takara, kusatsu, Japan) and primers bla_pec‐1_‐sacI‐up‐fwd/bla_pec_‐1‐KpnI‐down‐rev, and verified by sequencing (Eurofins). The resulting Δ*bla*_pec‐1_ mutants constructed in *P. versatile* CFPB6051 and *P. versatile* A73 strains were named CL1 and CL2, respectively.

### 2.3 In Vitro Growth Inhibition Assay

*Pectobacterium* strains were grown in TSB medium overnight at 28°C with shaking. The next day, the optical density (OD_600nm_) of the culture was adjusted to 0.15. The temperature of the molten LB agar was lowered to about 40°C (just before the agar solidified) and 25 mL of the LB agar was mixed with 1 mL of the bacteria to be tested in lawn culture. A spot of 5 μL of strain 6617 was then added to the inoculated plates, which were incubated at 30°C for 24–48 h before visualisation of the inhibition zone.

### 2.4 Potato Tuber Inoculations

Bacteria were plated on LB plates and incubated overnight at 28°C. A single colony was then used to inoculate 2 mL of LB medium, which was incubated overnight at 28°C with shaking at 120 rpm. This liquid culture (100 μL) was used to inoculate a 10% TSB agar plate, which was incubated overnight at 28°C. The grown bacterial layer was then suspended in 50 mM phosphate buffer (pH 7) and adjusted to OD_600nm_ = 1. For co‐inoculations, the strains involved were then mixed in equal volumes.

For each strain or mixture of strains, 10 potato tubers of the “charlotte” variety, previously surface sterilised with Javel 1.3% for 15 min and then rinsed, were inoculated. Each tuber was punctured with pipette tips containing 10 μL of the inoculum. The tip was left in the tuber. As a negative control, a pipette tip containing 10 μL of sterile phosphate buffer (50 mM) was inserted into a tuber. The inoculated tubers were placed on paper towels in plastic boxes; 50 mL of distilled water was poured into the bottom of the boxes, and the boxes were carefully sealed to maintain relative humidity above 90%. At 5 days post inoculation (5 dpi) at 26°C, the tubers were cut, and the entire macerated tuber tissue was collected and weighed. To count the bacterial load in the macerated tuber tissue, part of the macerate (50 to 300 mg) was weighed and suspended in 1 mL of sterile phosphate buffer (50 mM) and diluted in 10‐fold series, and 100 μL of the dilution 10^−5^ to 10^−7^ was spread on TSB plates and incubated for 24 h at 28°C. To analyse the fitness of the Δ*bla*_pec‐1_ mutant relative to the wild‐type strain, 100 individual colonies (per potato tuber analysed) were streaked onto CVP, TSB‐Amp, and TSB agar plates. Colonies that formed pits on CVP had the visual characteristics of *Pectobacterium* strains and grew on TSB‐Amp agar plates were defined as *P. versatile* wild‐type strains. Conversely, colonies that formed pits on CVP had the visual characteristics of *Pectobacterium* strains and were unable to grow on TSB‐Amp agar plates were defined as *P. versatile* Δ*bla*_pec‐1_ mutant derivatives.

To recover the bacteria from macerated tuber tissue for DNA or RNA extraction, the remainder of the macerated tissue was suspended in 7 mL of 50 mM phosphate buffer and stirred for 4 h at room temperature. The tubes were then left without shaking for 10 min to allow the excess starch to settle, and 2 mL was collected from the surface of the suspension and centrifuged at 10,000 g for 10 min. The resulting pellet was stored at −80°C until DNA or RNA extraction.

### 2.5 RNA Extraction and RT‐qPCR Analysis

To investigate the expression of *carA* and *bla*_pec‐1_, RNA was isolated using the PureLink RNA Mini Kit from Thermo Scientific, and 400 ng of RNA from 5 dpi macerated tuber tissue or bacteria grown overnight in LB medium was used to generate cDNA using the Thermo‐scientific maxima First Strand cDNA Synthesis Kit for RT‐qPCR with dsDNase. RT‐qPCR experiments were performed using SsoAdvanced Universal SYBR Green Supermix from BIO‐RAD. The housekeeping gene *gapA* was used to normalise the expression data for each gene of interest. The comparative quantitation method (ΔΔCt) was used to contrast the different treatments (Livak and Schmittgen 2001). Ct values quantify the number of PCR cycles required to amplify a template to a chosen threshold concentration, ΔCt values quantify the difference in Ct values between the target gene and the *gapA* gene for a given sample, and ΔΔCt values are used for the comparison between macerated tuber tissue and LB medium. Relative fold changes were calculated using 2^−ΔΔCt^.

### 2.6 DNA Extraction, DNA Amplification and Sequencing

DNA extraction was performed on 10 mL of the time zero inoculum, on 2 mL of 5 macerated potato tuber tissues for each inoculated strain mixture. Bacteria were pelleted by centrifugation and DNA extraction was performed using the genomic DNA purification Kit (Lucigen).

Amplification and sequencing were performed at MR DNA (www.mrdnalab.com, Shallowater, TX, USA). Either the *gap*A 376 partial gene sequence was amplified using PCR primers gapAF376 (GCCCGTCTCACAAAGA) and gapAR (TCRTACCARGAAACCAGTT) or the V3‐V4 region of the 16SrRNA gene was amplified using PCR primers 341F (CCTACGGGNGGCWGCAG) and 805R (GGACTACHVGGGTWTCTAAT) with barcodes on the forward primers. PCR was performed using the HotStarTaq Plus Master Mix Kit (Qiagen, USA) under the following conditions: 94°C for 3 min, followed by 28 cycles of 94°C for 30 s, 57°C for 40 s (for *gapA* amplicon) or 53°C for 40 s (for 16S amplicon) and 72°C for 1 min, followed by a final elongation step at 72°C for 5 min. Amplified PCR products were checked on a 2% agarose gel to determine the success of amplification and the relative intensity of bands. Multiple samples were pooled together in equal proportions based on their molecular weight and DNA concentration. Pooled samples were purified using calibrated Ampure XP beads and used to prepare an Illumina DNA library. Sequencing was performed on a MiSeq sequencer according to the manufacturer's guidelines. Sequence data were processed using the MR DNA analysis pipeline (MR DNA, Shallowater, TX, USA). Briefly, sequences were joined, depleted of barcodes, and sequences < 150 bp or with ambiguous base calls were removed. Reads were filtered based on Q‐score and expected error probability, and any read with an expected errors count greater than 1.0 was discarded.

### 2.7 Illumina Sequencing Analysis

After quality trimming, a total of 1,296,980 and 1,238,179 reads were obtained for 30 *gap*A and 20 16S analysed potato tubers (50 samples in total) assays respectively, with an average of 43,232 ± 8490 and 61,908 ± 18,049 reads per sample respectively.

To quantify the distribution of the *Pectobacterium* strains at time zero within the inoculum and at 5 dpi, SRP distribution analysis was performed at the strain level as previously described (Barny et al. 2024). Briefly, after *gap*A amplicon sequencing, reads were aligned to the *gap*A sequences of the 6 strains analysed ( *P. brasiliense* 6617, *P. brasiliense* 5381, *P. versatile* A73 wild‐type or Δ*bla*_PEC‐1_ and *P. versatile* 6051 wild‐type or Δ*bla*_PEC‐1_), using the nucleotide‐nucleotide Blastn tool (version 2.15.0+, e‐value threshold 10^−5^). Only reads with 100% full‐length identity were used (616,137 reads, 47.5% of the total reads).

Clustering, alignment and phylogenetic analysis of 16S gene fragments were performed using the QIIME2 (version 2023.5) (Bolyen et al. 2019) v.1.39.5 pipeline, dedicated to the study of microbial communities. Demultiplexing of 16S rDNA gene sequences and quality control were performed using DADA2 (Callahan et al. 2016). Taxonomic classification of each ASV was performed using SILVA (version 138) (Bokulich et al. 2018). The feature table of ASVs was rarefied to a sampling depth of 32,191 sequences per sample prior to downstream analyses.

## 3 Results

### 3.1 In Vitro, the β‐Lactamase Bla_PEC_ _−1_ Allows *P. versatile* to Resist Ampicillin or the Carbapenem Produced by *P. brasiliense*

**FIGURE 1:**
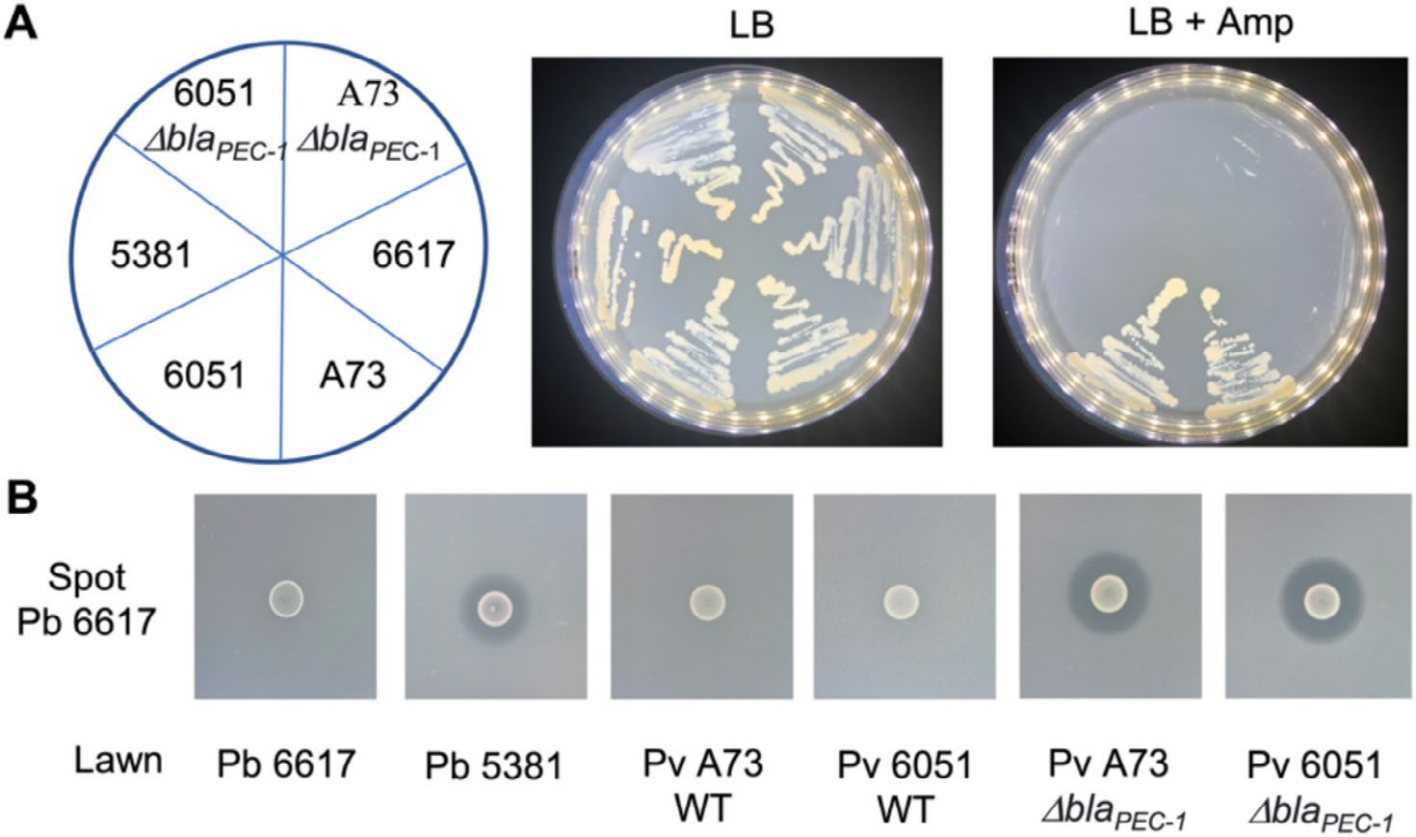
Spectrum of activity of Bla_PEC‐1_ β‐lactamase in vitro. (A) Growth of the strains shown in the left panel on LB agar (LB) and LB agar ampicillin (LB + Amp) plates. *Pectobacterium versatile* (Pv) strains: 6051 and A73; *P. brasiliense* (Pb) strains: 6617 and 5381. (B) Growth inhibition assays between the carbapenem‐producing strain Pb 6617 spotted and the lawn of strains Pb 6617, Pb 5381, Pb A73, PV 6051, Pv A73 Δ*bla*_PEC‐1_ mutant, and PV 6051 Δ*bla*_PEC‐1_ mutant.

To analyse the role of Bla_PEC‐1_, we constructed mutants deleted of the *bla*_PEC‐1_ gene in two *P. versatile* strains background, namely the type strain 6051 isolated from *Solanum tuberosum* and the strain A73 isolated from water. As expected, the two wild‐type strains were resistant to ampicillin and the two Δ*bla*_PEC‐1_ mutants became sensitive to this antibiotic, confirming that the ß‐lactamase Bla_PEC‐1_ confers resistance to the ß‐lactam ring of the penicillin class as previously observed by Royer et al. (2022) (Figure 1A). In contrast, the *P. brasiliense* 6617, which produces the carbapenem (5R)‐carbapen‐2‐em‐3‐carboxylic acid, was sensitive to ampicillin. This sensitivity indicates that the *carF* and *carH* genes required for resistance to the carbapenem produced are inefficient against ampicillin (McGowan et al. 1997). We also tested another *P. brasiliense* strain, 5381, which was sensitive to both ampicillin and to the carbapenem produced by *P. brasiliense* 6617. When the *P. versatile* strains A73 or 6051 were confronted in competition assays with the carbapenem‐producing strain *P. brasiliense* 6617, the two wild‐type strains were resistant, while the two Δ*bla*_PEC‐1_ mutants were sensitive, indicating that the β‐lactamase Bla_PEC‐1_ confers resistance to the simple carbapenem produced by *P. brasiliense* 6617 in addition to the penicillin class of ß‐lactamase rings (Figure 1B).

### 3.2 In the Absence of Carbapenem, Maceration Capacity and Fitness of *P. versatile* Δ*bla*_PEC_ _−1_ Mutants in Potato Tubers Are Not Altered Compared to WT Strains

**FIGURE 2:**
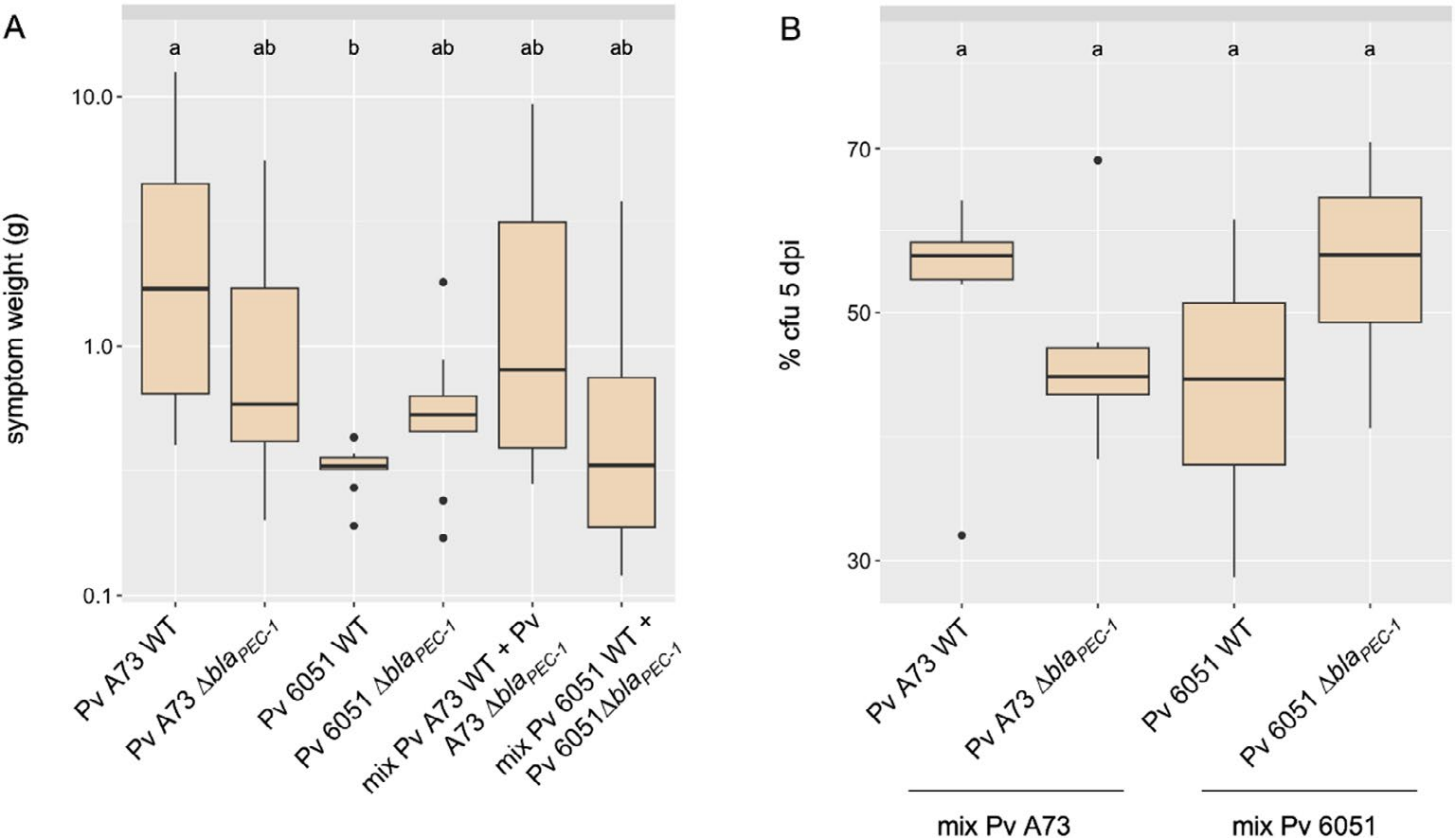
Maceration capacity and fitness of *Pectobacterium versatile* Δ*bla*_PEC‐1_ mutants. (A) Weight of macerated tuber tissue at 5 days post infection (dpi) for single species or mixed infections. The species or mixture inoculated on 10 potato tubers is indicated below each box plot. (B) The differential susceptibilities to ampicillin of *P. versatile* 6051/A73 wild‐type and 6051/A73 Δ*bla*_PEC‐1_ mutant strains were used to assess the fate of each strain within mixed infections. The box plots represent the bacterial abundance of each strain at 5 dpi. The result of the statistical analysis is indicated by the letters (a, b) at the top of the box plots. Bacterial mixtures sharing the same letter(s) are not statistically different from each other (*p* > 0.05 Kruskal Wallis followed by Dunn's test with the Bonferroni correction).

We then investigated whether deletion of the *bla*_PEC‐1_ gene altered the maceration capacity of the *P. versatile* strains in the absence of carbapenem. No difference in maceration capacity was observed between each wild‐type strain and its Δ*bla*_PEC‐1_ mutant derivative (Figure 2A). Strain A73 appeared to be slightly more aggressive than strain 6051, but this was only statistically significant when comparing the two wild‐type strains. We then analysed the fitness of each Δ*bla*_PEC‐1_ mutant when co‐inoculated with its wild‐type counterpart (Figure 2B). The different susceptibilities to ampicillin of the wild‐type *P. versatile* strains and their Δ*bla*_PEC‐1_ mutant derivatives (see Figure 1A) were used to quantify the respective presence of each strain in potato tubers at the end of the coinfection experiment (Figure 2B). Within the two mix‐inoculations, each wild‐type strain and its Δ*bla*_PEC‐1_ mutant derivative were present in the same proportion at the end of the experiment, indicating that the β‐lactamase Bla_PEC‐1_ neither affects nor enhances the fitness of either strain in potato tubers.

### 3.3 Coexistence of *P. versatile* Wild‐Type Strains With the Carbapenem‐Producing Strain *P. brasiliense* 6617 in Potato Tubers

**FIGURE 3:**
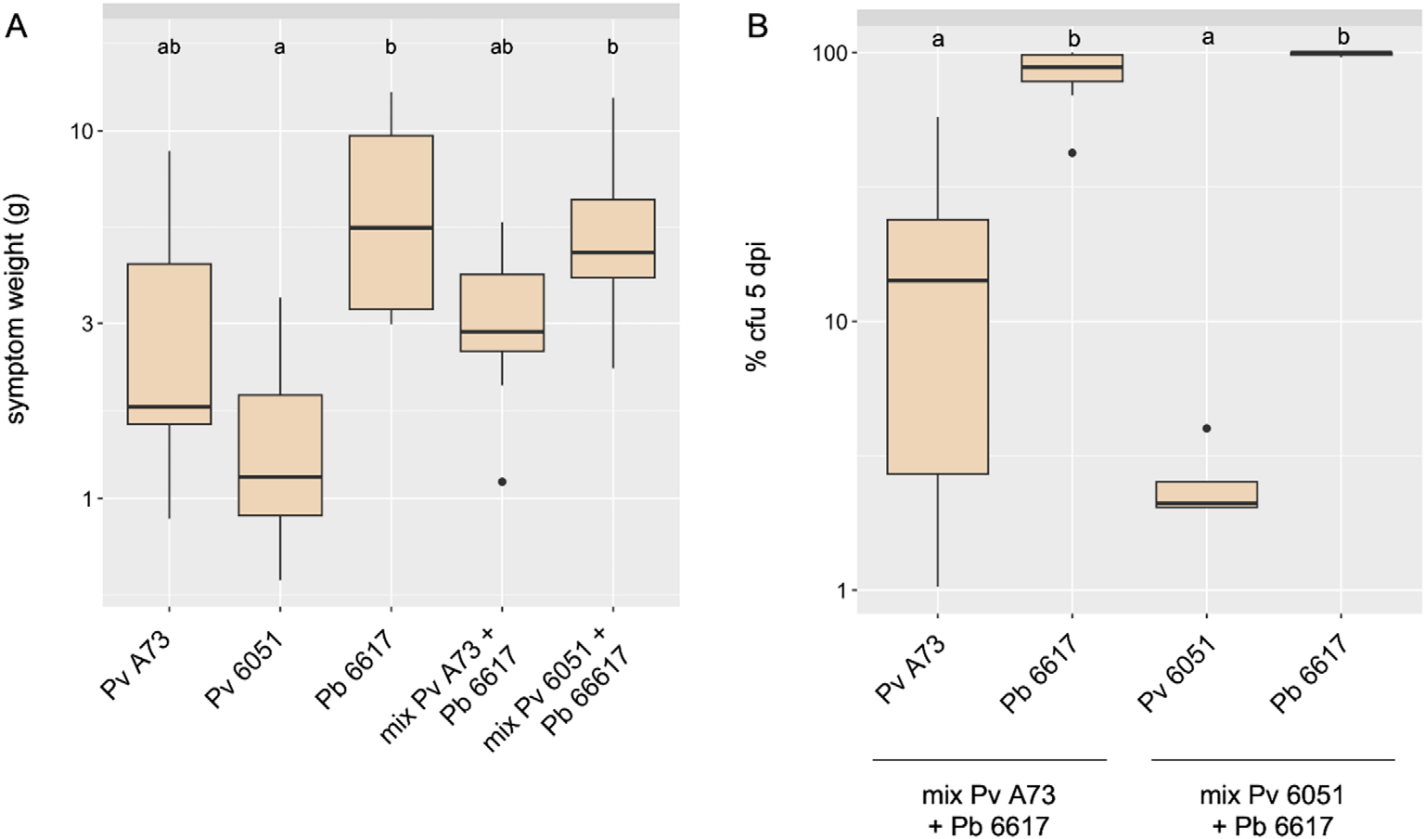
*In planta* competition between *Pectobacterium versatile* strain expressing Bla_PEC‐1_ and the *Pectobacterium brasiliense* strain expressing carbapenem. (A) Weight of macerated tuber tissue at 5 dpi for single species or mixed infections. The species or mixture inoculated on 10 potato tubers is indicated below each box plot. B: The differential susceptibility to ampicillin of *P. brasiliense* 6617 and *P. versatile* 6051/A73 wild‐type strains was used to assess the fate of each strain within mixed infections. The box plots represent the bacterial abundance of each strain (% cfu 5 dpi). The result of the statistical analysis is indicated by the letters (a, b) at the top of the box plots. The box plots with the same letter(s) are not statistically different from each other (*p* > 0.05 Kruskal Wallis followed by Dunn's test with the Bonferroni correction).

The in vitro resistance of the β‐lactamase‐producing wild‐type strains A73 and 6051 of *P. versatile* to the carbapenem produced by *P. brasiliense* 6617 prompted us to confront the strains *in planta*. To do this, we set up potato tuber infections with either a single infection of each strain or co‐infection between *P. brasiliense* and each of the wild‐type *P. versatile* strains (10 tubers per condition). There was considerable variation in maceration capacity (Figure 3A). Statistical analysis showed that *P. brasiliense* strain 6617 was more aggressive than *P. versatile* strain 6051. Strain A73 showed an intermediate level of aggressiveness that was not statistically different from either 6051 or 6617. Co‐inoculation of *P. brasiliense* 6617 with any *P. versatile* resulted in symptoms similar to a single inoculation with 6617 or A73, suggesting that the mixed inoculation does not affect disease progression. The different susceptibilities of *P. brasiliense* 6617 and *P. versatile* 6051/A73 to ampicillin (see Figure 1A) were used to quantify the respective presence of each strain in potato tubers at the end of the coinfection experiment. After co‐inoculation, *P. brasiliense* 6617 dominated the population at 5 dpi, consistent with its high level of virulence. Variability between inoculated tubers was important, but both *P. versatile* strains were detected at 5 dpi in 8 out of 10 and 5 out of 10 inoculated potato tubers for A73 and 6051, respectively. Overall, the mean recovery of *P. versatile* A73 was 14.5%, indicating its ability to coexist with *P. brasiliense* 6617. In contrast, *P. versatile* 6051 was strongly out competed by strain *P. brasiliense* 6617 with a mean recovery of 1.2% (Figure 3B).

### 3.4 No Association Was Found Between Commensal Abundance in Potato Soft Rot Symptoms and the Presence of the *P. versatile* *bla*_PEC_ _−1_ Gene

During infection of potato tubers, the important release of nutrients due to degradation of plant cell walls may allow the development of associated commensals (Kõiv et al. 2015). We tested whether the β‐lactamase Bla_PEC‐1_ affects commensal invasion. Using 16S metabarcoding, we compared the abundance and diversity of commensals in macerated tuber tissue at 5 dpi between 20 tubers infected with either the wild‐type strains or the Δ*bla*_PEC‐1_ mutants (5 tubers per condition). The presence of commensals was often limited and was less than 1% in 16 of the 20 tubers analysed, but a slightly higher proportion of associated commensals (from 2.1% to 14%) was observed in the 4 remaining potato tubers (Table 1). NMDS analysis indicated that commensal abundance was not statistically different between Δ*bla*_PEC‐1_ mutants and their wild‐type counterparts (Figure S1).

**TABLE 1:** Percentage of *Pectobacterium* and commensal at 5 dpi. Analysis was performed on each individual potato inoculated at 5 dpi (five replicates).

Strain inoculated	Potato	% *Pectobacterium*	% others
A73 WT	1	99.6	0.4
	2	99.6	0.4
	3	99.1	0.9
	4	99.6	0.4
	5	99.7	0.3
A73 *∆bla*_*PEC‐1*_	1	95.3	4.7
	2	99.4	0.6
	3	99.2	0.8
	4	97.8	2.2
	5	96.7	3.3
6051 WT	1	99.7	0.3
	2	99.7	0.3
	3	99.6	0.4
	4	99.7	0.3
	5	86.1	13.9
6051 *∆bla*_*PEC‐1*_	1	99.7	0.3
	2	99.6	0.4
	3	99.4	0.6
	4	99.7	0.3
	5	99.6	0.4

**FIGURE 4:**
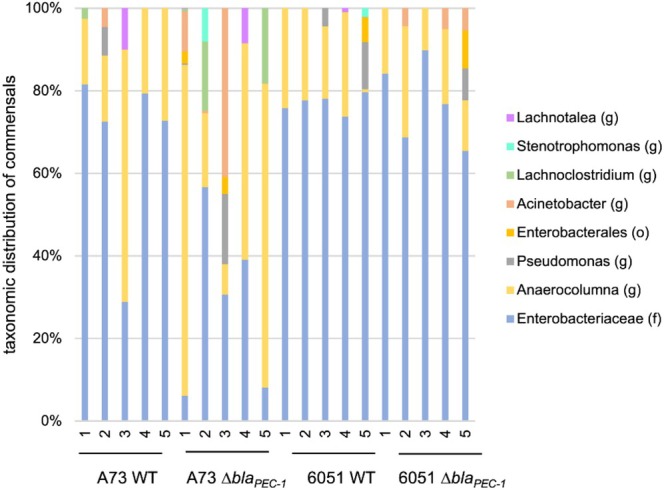
Commensal associated bacteria. Type of associated commensals in each analysed potato tuber at 5 dpi according to 16S barcode analysis. The inoculated strain and the potato number are indicated below each bar. The taxonomic assignment was made at the genus level (g) or higher when this was not possible (f: family, o: order).

We then analysed the nature of the commensals recovered in the macerated tuber tissue. The most common commensals in the 20 tubers analysed were unclassified *Enterobacteriaceae* and bacteria belonging to the genus *Anaerocolumna* (Figure 4). Other commensals varied in a stochastic way among potato tubers (Figure 4). In conclusion, the presence of commensals in macerated tuber tissue was variable between tubers and did not correlate with the presence of the *bla*_PEC‐1_ gene.

### 3.5 Expression of Genes Encoding Carbapenem and β‐Lactamase Within Potato Tuber

**FIGURE 5:**
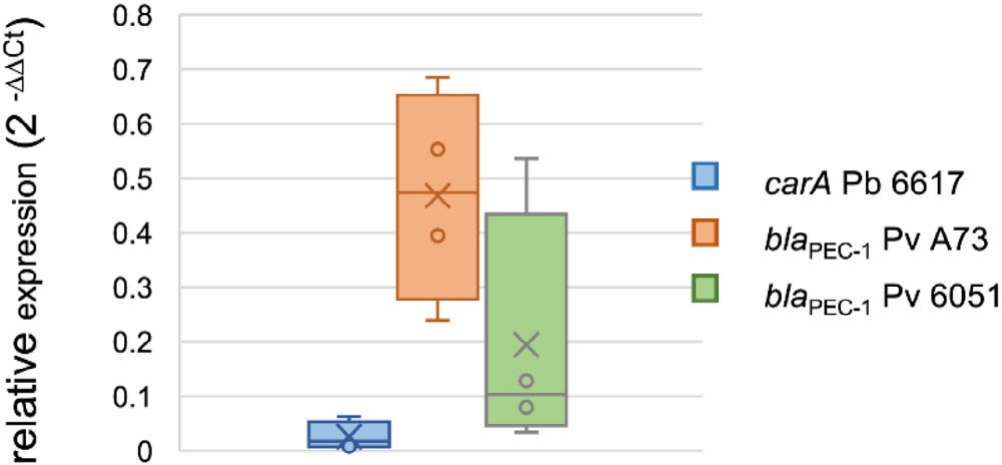
Relative expression of carbapenem and β‐lactamase encoding genes within potato tuber. 2^−ΔΔCt^ values were used to express the fold change in *car*A (Pb 6617 strain) and *bla*_PEC‐1_ (Pv A73 and 6051 strains) gene expression in macerated tuber tissue relative to expression in LB medium. A relative fold change of less than 1 indicates repression *in planta* compared to LB conditions in vitro. Expression data were normalised using the housekeeping gene *gap*A as a reference gene.

To compare the level of expression of the genes involved in the synthesis of carbapenem or Bla_PEC‐1_ β‐lactamase during infection of potato tubers and LB synthetic medium, we set up a mono‐infection experiment with either the *P. brasiliense* strain 6617 or the *P. versatile* strains 6051 and A73. The expression levels of the *bla*_PEC‐1_ and *carA* genes involved in the production of Bla_PEC‐1_ β‐lactamase and carbapenem respectively were quantified by RT‐PCR (McGowan et al. 1997; Royer et al. 2022). Large differences between potato tubers were observed. However, the *carA* gene of *P. brasiliense* 6617 was consistently strongly repressed in potato tubers compared to LB medium (Figure 5). Similarly, the *bla*_PEC‐1_ gene was also repressed in potato tubers compared to LB medium but to a lesser extent than that observed for the *carA* gene.

### 3.6 *P. versatile* *bla*_PEC_ _−1_ Gene Is Required During Co‐infection With Carbapenem‐Producing *P. brasiliense* to Protect Another *P. brasiliense* Strain Sensitive to Carbapenem

**FIGURE 6:**
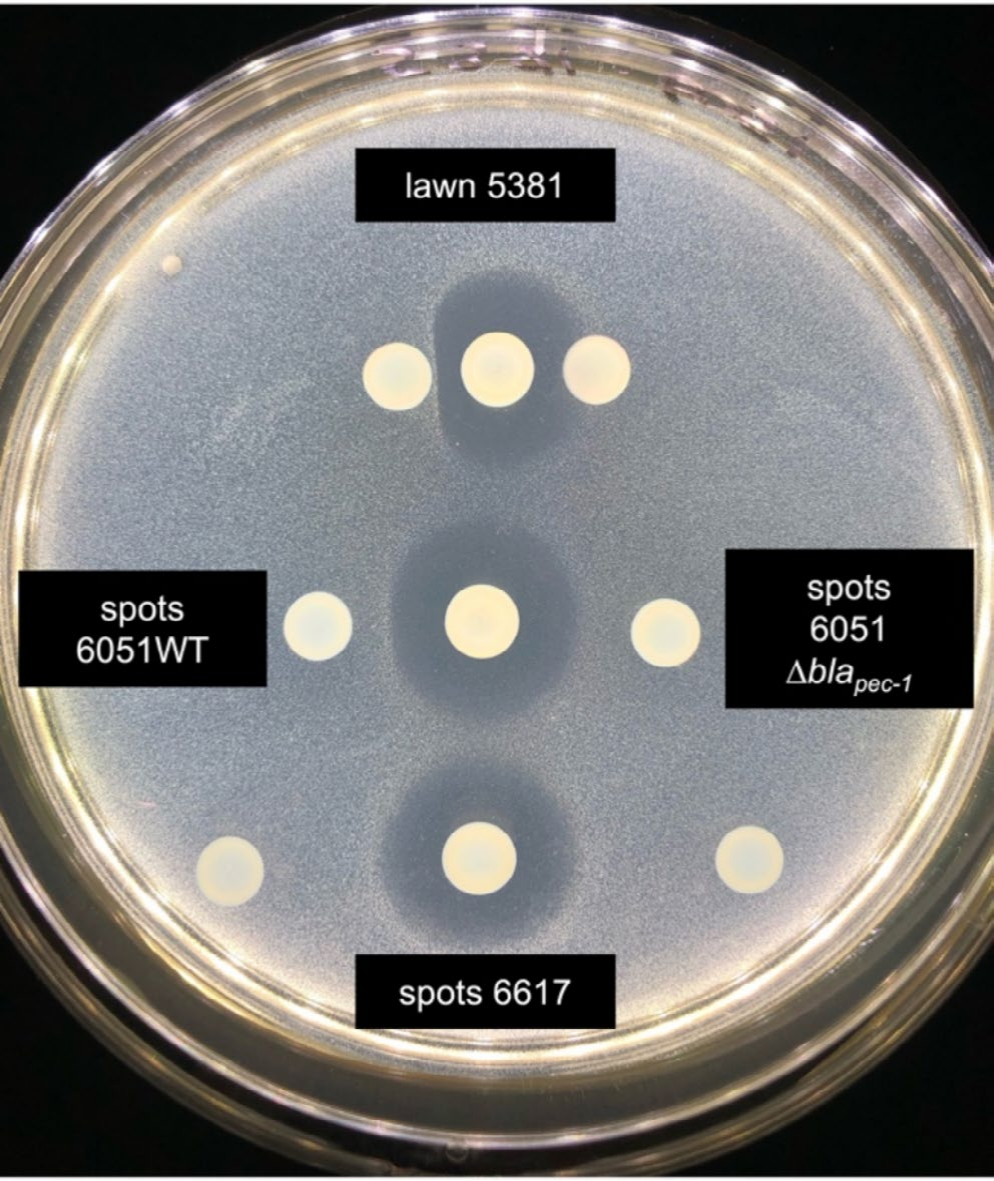
In vitro, the β‐lactamase Bla_PEC‐1_ confers protection against carbapenem. The *Pectobacterium brasiliense* 5381 strain grown in the lawn is sensitive to the carbapenem produced by the *P. brasiliense* 6617 strain spotted in the center of the Petri dish. The wild‐type *Pectobacterium versatile* strain 6051 spotted on the left protects the sensitive strain only when the spot is in proximity (compare upper and lower spots). None of the spots of the 6051 Δbla_PEC‐1_ mutant of *P. versatile* on the right confers protection.

During infection, mixtures of SRP spp. may account for up to 20% of the observed symptoms (Degefu 2021; de Werra et al. 2021; Ge et al. 2021; Motyka‐Pomagruk et al. 2021). We therefore wondered whether the *P. versatile* β‐lactamase Bla_PEC‐1_ could protect other *Pectobacterium* strains from the carbapenem produced by the *P. brasiliense* strain 6617 (Shyntum et al. 2019). In vitro, the carbapenem‐sensitive strain *P. brasiliense* 5381 was exposed to the carbapenem‐producing strain 6617 in the vicinity of either the *P. versatile* β‐lactamase‐producing strain 6051 or its Δ*bla*_PEC‐1_ mutant derivative. The presence of the *P. versatile* strain 6051, but not of the 6051 Δ*bla*_PEC‐1_ mutant, protected the sensitive strain 5381 from the toxic effect of *P. brasiliense* strain 6617 (Figure 6). This indicates that, in vitro, the β‐lactamase Bla_PEC‐1_ secreted by the WT strain of *P. versatile* is able to degrade the carbapenem produced and secreted by *P. brasiliense* 6617 efficiently enough to protect the sensitive strain 5381 when this latter strain is close enough to the β‐lactamase BlaPEC‐1 produced by *P. versatile*.

**FIGURE 7:**
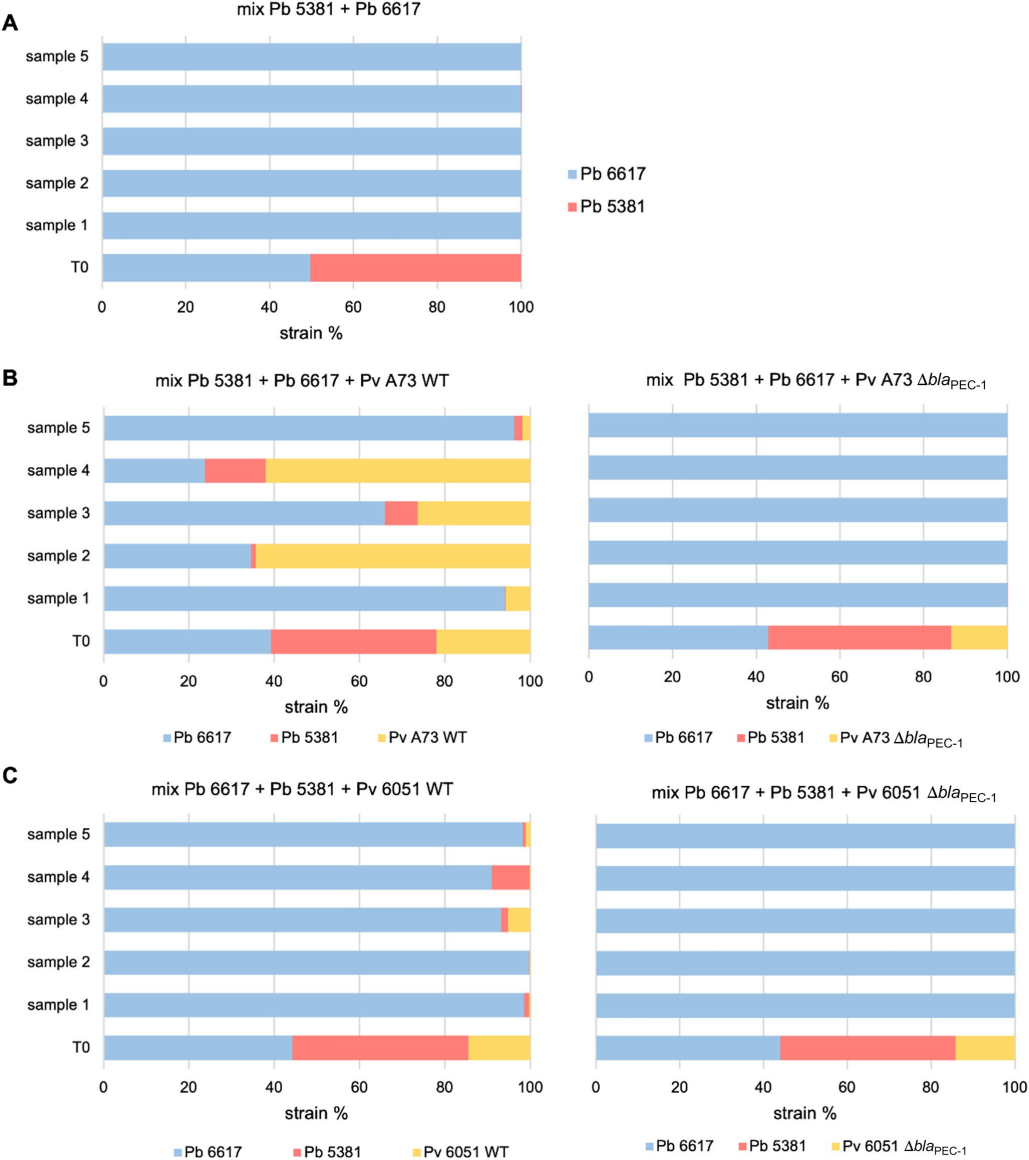
Fate of SRP consortium in mixed inoculation on potato tubers.

We then ask whether the protection conferred by *P. versatile* β‐lactamase‐producing strains is also observed *in planta*. Potato tuber co‐infection experiments were set up with (1) the carbapenem‐producing *P. brasiliense* strain 6617, (2) the carbapenem‐sensitive *P. brasiliense* strain 5381, and (3) either the wild‐type *P. versatile* strains A73 or 6051 or their Δ*bla*_PEC‐1_ mutant derivatives. As a control, we also tested whether the carbapenem‐sensitive strain 5381 is maintained when co‐inoculated alone with the carbapenem‐producing strain 6617. The proportion of each strain at the beginning and end of the experiment was assessed by Illumina sequencing of the *gap*A discriminating gene marker (Barny et al. 2024). When the carbapenem‐sensitive strain 5381 was co‐inoculated alone with the carbapenem‐producing strain 6617, it was outcompeted, and the only strain recovered at the end of the experiment was the carbapenem‐producing strain 6617 (Figure 7A). Similarly, in the mixture containing 6617 + 5381 and either the A73 Δ*bla*_PEC‐1_ mutant or the 6051 Δ*bla*_PEC‐1_ mutant, only the carbapenem‐producing 6617 was recovered at the end of the experiment (Figure 7B,C). In contrast, in the presence of the wild‐type *P. versatile* strains A73 or 6051, both the *P. versatile* strains and the sensitive strain 5381 were detected at varying levels at the end of the experiment in 9 out of the 10 potato tubers (Figure 7B,C). This indicates that the β‐lactamase Bla_PEC‐1_ protects both the *P. versatile* strain itself and the carbapenem‐sensitive strain 5381 from the toxic effect of the carbapenem produced by 6617 as observed in vitro. The protection conferred by strain 6051 was less than that conferred by strain A73, consistent with its weaker ability to compete with strain 6617. However, no correlation was observed between the proportion of *P. versatile* wild‐type strains and the proportion of the carbapenem‐sensitive strain 5381 recovered within the macerated tuber tissue at 5 dpi (Figure 7B,C). This lack of correlation is likely explained by spatial effects within the macerated tuber tissue, since in vitro rescue of the carbapenem‐sensitive strain 5381 is highly dependent on spatial effects as observed in vitro (Figure 6).

The inoculated (T0) is compared with the mix observed at 5 dpi after for 5 independent potato tubers. The strains involved in each consortium are followed by analysis of the *gap*A barcode (Barny et al. 2024). Carbapenem‐producing strains are shown in blue, carbapenem‐sensitive strains are shown in red, and β‐lactamase‐producing strains are shown in yellow. A: mix between the carbapenem‐producing *P. brasiliense* 6617 strain and the carbapenem‐sensitive *P. brasiliense* 5381 strain. B: mix between the carbapenem‐producing *P. brasiliense* 6617 strain, the carbapenem‐sensitive *P. brasiliense* 5381 strain, and either the *P. versatile* A73 WT strain expressing the β‐lactamase Bla_PEC‐1_ or its Δ*bla*_PEC‐1_ mutant derivative. C: mix between the carbapenem‐producing *P. brasiliense* 6617 strain, the carbapenem‐sensitive *P. brasiliense* 5381 strain, and either the *P. versatile* 6051 WT strain expressing the β‐lactamase Bla_PEC‐1_ or its Δ*bla*_PEC‐1_ mutant derivative.

## 4 Discussion

The β‐lactamase Bla_PEC‐1_ is the closest relative of Bla_TEM‐1_, a class A2b β‐lactamase that is plasmid‐borne and widespread in clinical enterobacterales. Bla_PEC‐1_, like Bla_TEM‐1_, cleaves β‐lactam rings of the penicillin family but is inefficient on clinically used carbapenems such as meropenem or imipenem (Royer et al. 2022). Here we have shown, using in vitro competition assays, that the β‐lactamase Bla_PEC‐1_ is nevertheless efficient in inactivating the simplest of the carbapenem molecules produced by *P. brasiliense* . This suggests that the inability of Bla_PEC‐1_ to inactivate imipenem and meropenem is probably due to the steric hindrance provided by the C‐6 side chain of these clinically useful carbapenems. It remains to be determined whether Bla_TEM‐1_, which is clinically widespread around the world and is the β‐lactamase closest to Bla_PEC‐1_, is also capable of cleaving this simplest carbapenem molecule.

We then analysed the role of the *P. versatile* β‐lactamase Bla_PEC‐1_ in the context of plant infection in the absence of antibiotic pressure. *In planta*, during mono‐infection experiments, we could not detect a fitness cost associated with the presence of Bla_PEC‐1_ as wild‐type and bla_PEC‐1_‐deleted strains multiplied similarly in macerated tuber tissue. The fitness cost of a resistance determinant has been shown to depend in part on its genetic localisation, such as a chromosomal mutation or a plasmid acquisition (Vanacker et al. 2023). The lack of fitness cost associated with the *bla*_PEC‐1_ gene may be partly due to the chromosomal location of this gene within the *P. versatile* genomes (Royer et al. 2022). It has also been shown that the fitness costs of antibiotic resistance genes are lower for β‐lactamases compared to mutations in core genes which have a more pleiotropic effect that could be deleterious in the absence of antibiotic pressure (Vanacker et al. 2023). The absence of detectable fitness costs for chromosomally encoded β‐lactamases in the absence of antibiotic pressure probably explains the wide distribution of these enzymes within natural communities. For example, in remote, undisturbed Alaskan soils with no antibiotic selection pressure, 5% of bacterial genomes carry diverse β‐lactamase genes that are readily expressed in *Escherichia coli* (Allen et al. 2009). Similarly, permafrost bacteria represent a vast reservoir of antibiotic resistance genes, among which β‐lactamase genes have been observed at high frequencies (Rigou et al. 2022).

Whether β‐lactamases play a real role in antibiotic resistance in natural environments has been debated due to their low concentration (Davies et al. 2006; Aminov 2009). In vitro Bla_PEC‐1_ expression levels were low compared to that of Bla_TEM‐1_ (Royer et al. 2022), suggesting that the role of Bla_PEC‐1_ during infection may only be a signalling role as previously proposed (Romero et al. 2011). Quantification of *bla*_PEC‐1_ expression within the potato tuber showed that *bla*_PEC‐1_ is repressed compared to its expression level in LB medium. However, *P. versatile* β‐lactamase can be continuously secreted into the environment through membrane vesicles (Jonca et al. 2021) and this could potentialise its action. At the same time, the expression level of the *carA* gene, which is involved in the biosynthesis of the carbapenem by strain 6617, is also strongly repressed within macerated tuber tissue at 5 dpi. This is consistent with the dramatic repression of carbapenem expression observed in vitro in an anaerobic medium (McGowan et al. 2005; Shyntum et al. 2019). Consistent with anaerobiosis, we found that commensals of the genus *Anaerocolumna*, a strictly anaerobic genus (Ueki et al. 2016), are systematically found associated with tuber rot symptoms. Surprisingly, despite the strong repression of *car*A in potato tubers, we found that a carbapenem‐sensitive strain was strongly outcompeted and could not be recovered when mixed inoculated with the carbapenem‐producing strain. This indicates that carbapenem production is still efficient during infection, although its expression is severely repressed compared to LB medium. The efficacy of carbapenem in the potato tuber was also unexpected because the carbapenem produced is a highly unstable molecule (Parker et al. 1982). This shows that lower expression levels than the one observed in vitro and instability are not sufficient to rule out functionality and highlights the need for careful analysis to understand the effect of antibiotic production in natural environments.

When a *P. versatile* β‐lactamase producing strain was added to the consortium, this third strain was able to protect the carbapenem‐sensitive strain from the deleterious effect of the carbapenem‐producing strain. This protection was space dependent and was only observed in vitro when the 3 strains were in proximity. *In planta*, the protection was effective even when the *P. versatile* strain was a minority within the tuber rot symptoms and the level of protection, exemplified by the maintenance of the carbapenem sensitive strain, did not correlate with the proportion of each strain (protective, sensitive and antibiotic producing) within the symptoms. This probably highlights the formation of specific micro‐niches within the macerated tuber tissue. This is consistent with the fact that stochastic positioning in the initial phase of colonisation can be a critical factor that determines the subsequent population dynamics (Von Bronk et al. 2017).

Within the SRP species complex, *P. versatile* occupies a special place as it is the most abundant SRP species, both on symptomatic or asymptomatic crops and in the environment, highlighting its ecological success (Portier et al. 2020; Ben Moussa et al. 2022; Smoktunowicz et al. 2022). Among SRP species, *P. versatile* is also the only species that harbours the β‐lactamase Bla_PEC‐1_ at high frequency (Royer et al. 2022), suggesting that Bla_PEC‐1_ may be involved in its ecological success. Here we showed that Bla_PEC‐1_ exerts a true β‐lactamase function, allowing *P. versatile* to persist in the face of a carbapenem‐producing species. *P. versatile* is not considered to be a major pathogen of the SRP species complex, so the β‐lactamase Bla_PEC‐1_ may allow *P. versatile* to take advantage of aggressive carbapenem‐producing SRP species during infection in order to proliferate during disease progression. Whether Bla_PEC‐1_ also plays a role in other aspects of the lifestyle of *P. versatile* as it spreads in the environment remains to be determined. As *P. versatile* is often associated as a companion species with species responsible for epidemic outbreaks (van der Wolf et al. 2021), our results also highlight the role of the *P. versatile* β‐lactamase in maintaining strain diversity within the SRP complex. The maintenance of SRP diversity is crucial, allowing the regular emergence of new epidemic clones, such as those regularly observed on the potato host (Jonkheer et al. 2021). Beyond the SRP species complex, the high occurrence of different β‐lactamases in natural pristine environments has recently been described (Lima‐Bittencourt et al. 2007; Pedroso et al. 2017; Fonseca et al. 2018; Rigou et al. 2022). The work carried out here sheds light on the role played by β‐lactamases in maintaining the diversity of β‐lactam‐susceptible strains in the environment.

## Author Contributions

**Camille Lorang:** investigation, methodology, writing – review and editing. **Pierre‐Yves Canto:** investigation, methodology, writing – review and editing, visualization. **Erwan Gueguen:** supervision, writing – review and editing. **Jacques Pédron:** conceptualization, supervision, formal analysis, writing – review and editing, visualization. **Marie‐Anne Barny:** conceptualization, supervision, formal analysis, funding acquisition, writing – original draft, writing – review and editing, investigation.

## Ethics Statement

All applicable local, national and international regulations and conventions, as well as normal scientific ethical practices, were followed in the preparation of this work.

## Conflicts of Interest

The authors declare no conflicts of interest.

## Supporting information


**Data S1.** Supporting Information.

## Data Availability

The data shown in this paper are available within the article and Supporting Information.
