# Supplementary material for: Pectobacterium versatile β‐Lactamase Contributes to Soft Rot Pectobacteriaceae (SRP) Community Diversity During Potato Infection
Source: Environ Microbiol Rep. 2025 May 26;17(3):e70111. doi: 10.1111/1758-2229.70111 (PMC12104563; doi:10.1111/1758-2229.70111)
Supplement: Supplementary file 1 — Data S1. Supporting Information. [file EMI4-17-e70111-s001.docx]

**Supplementary Figure and tables**


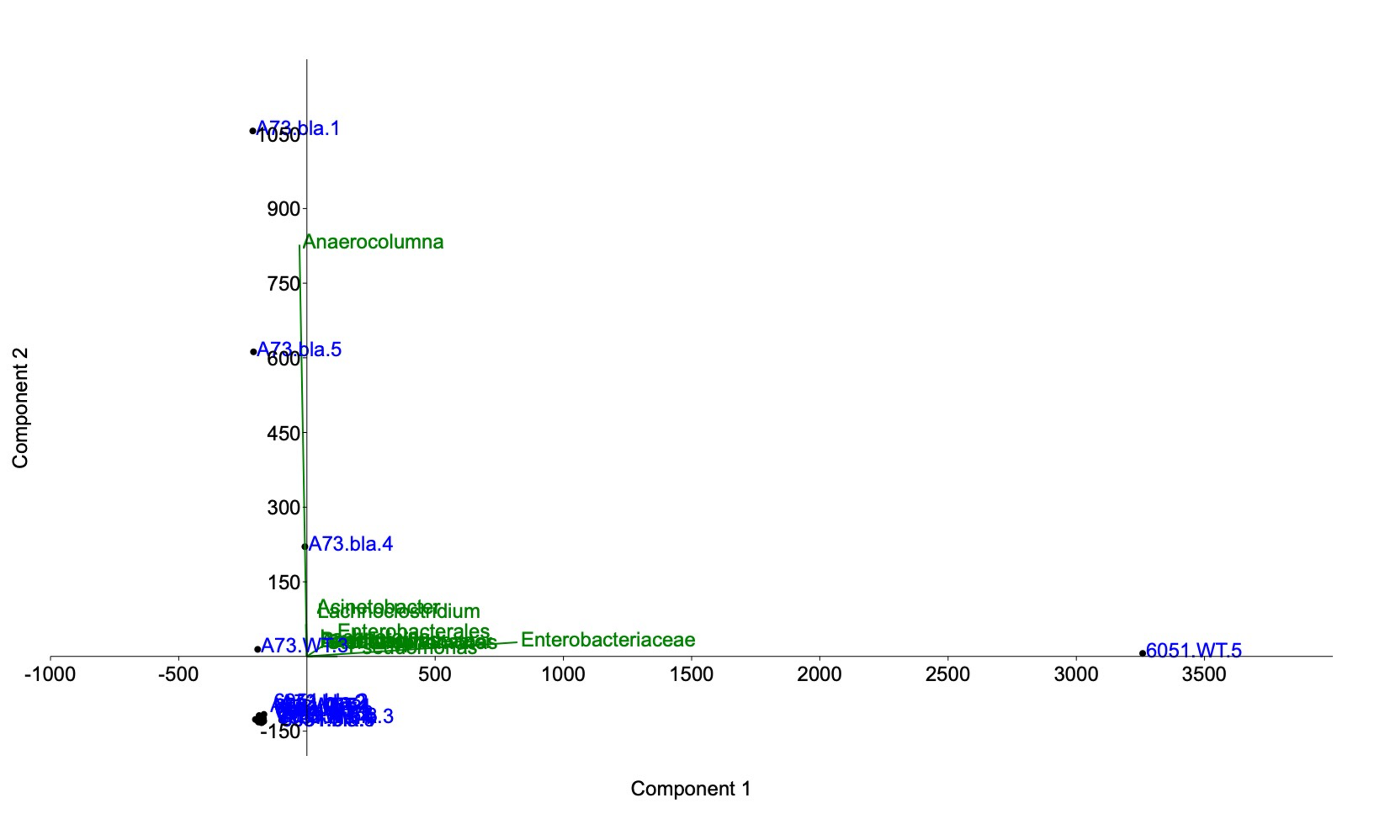


**Figure S1: Principal components analysis of commensals.**

Principal component analysis was performed on the abundance matrix of commensal bacterial genera, 5 replicates per strain. Only 5 samples out of 20 (in blue) stand out, without forming a coherent group. For these samples, 2 variables (in green) are explanatory (genera *Anaerocolumna* and *Enterobacteriaceae*).

**Table S1: Bacterial strains and plasmids**

| Bacterial strains  and plasmids | Description | Source and  reference |
| --- | --- | --- |
| **Strains** |  |  |
| *Escherichia coli* K12 |  |  |
| DH5α | *supE44 lacU169 (*Φ*80lacZ*∆ M15) *hsdR17 (rK mK ) recA1 endA1 gyrA96 thi-1 relA1* | Lab collection |
| DH5α λpir | λpir phage lysogen of DH5α | E. Gueguen |
| MFD*pir* | *RP4-2-Tc::(∆Mu1::aac(3)IV-∆aphA-·∆nic35-∆Mu2::zeo) ∆dapA::erm-pir) ∆recA* | E. Gueguen |
|  |  |  |
| *Pectobacterium versatile* | | |
| CFBP6051 | Type strain, isolated from *solanum tuberosum* in Netherland, Amp^R^ | CIRM-CFBP (Portier *et al.*, 2019) |
| CL1 | CFBP6051Δ*bla*_pec-1_, Amp*S* | This study |
| A73 | Isolated from river water, Amp^R^ | Lab collection (Ben Moussa *et al.*, 2022) |
| CL2 | A73 Δ*bla*_pec-1_, Amp*S* | This study |
| *Pectobacterium brasiliense* | | |
| CFBP6617 | Type strain, isolated from *solanum tuberosum* in Brazil, Amp^S^ | CIRM-CFBP (Liu and Filiatrault, 2020) |
| CFBP5381 | Isolated from *solanum tuberosum* in Algeria, Amp^S^ | CIRM-CFBP (Liu and Filiatrault, 2020) |
| **Plasmids** |  |  |
| pRE112 | Suicide vector for allelic exchange, Cm^R^, *sacB*, *oriT* RP4, *ori*R6K | E. Gueguen (Edwards *et al.*, 1998) |
| pCL1 | pRE112- Δ*bla*_pec-1_ (∆CL1_sequences à déposer), Cm^R^ | This study |
| pCL2 | pRE112- Δ*bla*_pec-1_ (∆CL2_ séquences à déposer), Cm^R^ | This study |

**Table S2: Oligonucleotides used in this study**

| **Oligonucleotide** | Sequence (5’-3’) | Use |
| --- | --- | --- |
| **L762** | gttattggtgcccttaaacg | Primers to check the correct cloning into pRE112. Amplification of the cloned region send to sanger sequencing (eurofin) |
| **L763** | gcatccaacgccattcatgg |  |
| **gapAF376** | GCCCGTCTCACAAAGA | Amplification of *gapA* 376bp partial gene sequence for Illumina sequencing |
| **gapAR** | TCRTACCARGAAACCAGTT |  |
| **341F** | CCTACGGGNGGCWGCAG | Amplification of the V3-V4 region of the 16SrRNA gene for iIlumina sequencing |
| **805R** | GGACTACHVGGGTWTCTAAT |  |
| **bla_pec-1_-sacI-up-fwd** | AACTGCATGaattcccgggagagctcttcg | Amplification of the upstream 0.5-kb DNA fragments of *P. versatile A73 and CFBP6051 for* cloning into pRE112 |
| **bla_pec-1_-up-rev** | gTTACCAGTGCTTGAAATGTTGCATattaa |  |
| **bla_pec-1_-down-fwd** | ATGCAACATTTCAAGCACTGGTAAcaatca | Amplification of the downstream 0.5-kb DNA fragments of *P. versatile A73 and CFBP6051* and cloning into pRE112 |
| **bla_pec-1_-KpnI-down-rev** | gatcccaagcttcttctagaggtaccagat |  |
| ***bla*_PEC-1-_fwd** | AAA GAT ACT TAC TGT CGC C | RT-qPCR - *bla*_PEC-1_ gene |
| ***bla*_PEC-1_rev** | TAT CCG CAA TAA ACC AGC C |  |
| ***carA-*fwd** | AGT GTC AGA TGG AAA GAG GA | RT-qPCR-*carA* gene |
| ***carA-*rev** | GAC ATG AAC CGG CGA GAA |  |
| ***gapA-*fwd** | GTA TCG GCC GTA TTG TTT T | RT-qPCR-*gapA* gene |
| ***gapA-*rev** | TTC GTT CCA CTT CAG GTT |  |
